# Biomediated control of colloidal silica grouting using microbial fermentation

**DOI:** 10.1038/s41598-023-41402-z

**Published:** 2023-08-30

**Authors:** Michael G. Gomez, Samantha T. Muchongwe, Charles M. R. Graddy

**Affiliations:** 1 Department of Civil and Environmental Engineering, University of Washington, Seattle, WA 98195 USA; 2 Department of Microbiology and Molecular Genetics, University of California, Davis, CA 95616 USA

**Keywords:** Civil engineering, Applied microbiology, Biogeochemistry

## Abstract

Colloidal silica grouting is a ground improvement technique capable of stabilizing weak problematic soils and achieving large reductions in soil hydraulic conductivities for applications including earthquake-induced liquefaction mitigation and groundwater flow control. In the conventional approach, chemical accelerants are added to colloidal silica suspensions that are introduced into soils targeted for improvement and the formation of a semi-solid silica gel occurs over time at a rate controlled by suspension chemistry and in situ geochemical conditions. Although the process has been extensively investigated, controlling the rate of gel formation in the presence of varying subsurface conditions and the limited ability of conventional methods to effectively monitor the gel formation process has posed practical challenges. In this study, a biomediated soil improvement process is proposed which utilizes enriched fermentative microorganisms to control the gelation of colloidal silica grouts through solution pH reductions and ionic strength increases. Four series of batch experiments were performed to investigate the ability of glucose fermenting microorganisms to be enriched in natural sands to induce geochemical changes capable of mediating silica gel formation and assess the effect of treatment solution composition on pH reduction behaviors. Complementary batch and soil column experiments were subsequently performed to upscale the process and explore the effectiveness of chemical, hydraulic, and geophysical methods to monitor microbial activity, gel formation, and engineering improvements. Results demonstrate that fermentative microorganisms can be successfully enriched and mediate gel formation in suspensions that would otherwise remain highly stable, thereby forgoing the need for chemical accelerants, increasing the reliability and control of colloidal silica grouting, enabling new monitoring approaches, and affording engineering enhancements comparable to conventional colloidal silica grouts.

## Introduction

Colloidal silica grouting is an environmentally conscious ground improvement technique capable of improving the engineering properties of rock and soil for applications including earthquake-induced liquefaction mitigation, groundwater flow control, and rock fracture sealing^1–11^. The process can be initiated by supplying a low viscosity suspension of non-porous, spherical silica nanoparticles to soils, with the formation of a silica gel occurring over time at a rate controlled by initial suspension chemistry^3,12^. Resulting colloidal silica gels can reduce soil hydraulic conductivities by plugging soil pore space and alter soil mechanical behaviors through the restraint of soil volumetric tendencies during shearing and the addition of a modest tensile strength^9,13,14^. Colloidal silica grouts offer some unique benefits over other permeation grouting technologies including: (i) the ability to apply grouts passively using existing groundwater gradients due to the low initial viscosity of colloidal silica suspensions^3,15^, (ii) the ability to modulate gel formation rates over large timescales (i.e., 0 to > 100 days)^13,16^(iii) the environmentally benign chemical properties of colloidal silica which can minimize environmental impacts when compared to other synthetic grouting materials such as polyurethanes^17,18^, and (iv) the ability of developed colloidal silica gels to remain chemically stable over long time periods following application^16,19^.

Numerous studies have examined the stability of colloidal silica suspensions and the time-dependent formation of silica gels for a diverse range of applications spanning from soil improvement to food processing^11,19,20^. Collectively, these studies have demonstrated that the time required to achieve gel formation can be controlled by varying the composition of colloidal silica suspensions, including through differences in initial pH, ion concentrations, colloidal silica concentrations, and the size of included colloids^1,3,12,21,22^. The sensitivity of colloidal silica suspensions to changes in chemistry results primarily from the presence of silanol (SiOH) functional groups on the surface of silica nanoparticles, which can be easily manipulated through changes in pH (i.e., H^+^ ions) and cation/anion concentration^19^. Under more acidic conditions, these surface groups can remain increasingly protonated with a more positive apparent surface charge, however, under more alkaline conditions the deprotonation of surface groups results in a more negative apparent surface charge^23,24^. Similar to pH changes, colloidal silica surface groups also exhibit sensitivity to changes in surrounding ion concentrations. For example, cations such as sodium (Na^+^) can complex with these surface groups, thereby allowing the apparent charge of surface groups to be effectively neutralized. Although highly complex, the interactions observed between silica colloids are similar to those described by Derjaguin, Landau, Verwey, and Overbeek (DVLO) theory^25^. When colloidal surface groups remain either highly negatively charged or highly positively charged, electrostatic repulsion between colloids remains high and the suspension can remain stable with the retention of a low solution viscosity ideal for transport during grout injections^3^. However, as colloidal surface groups become progressively neutralized either through pH changes or ion additions, electrostatic repulsion can be minimized and van der Waals attraction between colloids can enable the formation of siloxane bonds (Si–O–Si) between SiOH^-^ surface groups^26^ leading to polymerization of the nanoparticles and a consequential increase in the viscosity of the suspension and the eventual formation of a semi-solid silica gel.

Although injected suspension chemistries can be modified to alter gel formation times, such compositions must be carefully designed to be compatible with grout delivery rates and subsurface geochemical conditions. Past field trials have exemplified the challenges associated with adjusting gelation rates via initial chemical compositions, particularly when subsurface soils and groundwater are acidic and/or contain high dissolved ion concentrations^16,27^. Under such conditions, gelation can proceed more rapidly than expected thereby clogging injection wells and preventing effective distribution of suspensions to targeted soil volumes. Conversely, when supplied accelerants are insufficient or subsurface conditions differ from initial expectations, suspensions can also fail to gel, resulting in minimal engineering improvements. Furthermore, traditional in situ monitoring methods such as cone penetration and shear wave velocity testing have been shown to have limited ability to resolve changes in gel formation^16^ with monitoring of the process largely limited to assessment of solution delivery^28^. While the conventional abiotic grouting process requires careful titration of suspension chemistries prior to injections to ensure controlled gelation, the use of microbial activity to control process timing may provide a superior alternative to chemical additions with the potential to induce rapid, predictable chemical changes following injections and the meditation of gel formation for otherwise highly stable suspensions. For colloidal silica grouts, microbial processes which can alter surrounding solution pH values and generate increases in charged solution species can enable mediation of gel formation through the neutralization of colloidal silica surface charge. Although other processes could be used to mediate gel formation, microbial glucose fermentation may be an ideal pathway, requiring no oxygen and enabling large reductions in solution pH with associated increases in ionic strength^29^. Previous studies have used microbial glucose fermentation to mediate other soil improvement processes including the formation of calcium alginate gels through the dissolution of calcium carbonate minerals previously established via ureolytic biocementation and the release of calcium ions^30^. In the study by Cheng et al. (2019)^30^, fermentative activity was established by inoculating soils with a mixed microbial culture obtained from activated sludge and provided in solutions containing alginate and glucose (pH_initial_ = 7.5). Although using a different microbial pathway, Maclachlan et al. (2013)^31^ demonstrated that microbial urea hydrolysis could be used to mediate the gelation of initially acidic colloidal silica grouts through increases in solution ionic strength and pH. In this study, mediation of the process via ureolysis was found to achieve a more uniform gel structure, faster gelation, and a higher gel shear strength when compared to gels formed using chemical accelerants, albeit with the generation of aqueous ammonium resulting from urea hydrolysis. Collectively, outcomes from both studies suggest that microbial glucose fermentation activity can provide a viable pathway to control the gelation of colloidal silica grouts. Although similar in principle to the work by Maclachlan et al. (2013)^31^, the use of glucose fermentation activity was expected to address limitations related to the use of microbial ureolysis by providing a method capable of improving soils under more acidic conditions and eliminating the production of ammonium by-products.

Fermentation pathways are varied and widely distributed among microorganisms which can ferment supplied carbohydrates (i.e., glucose) under anaerobic conditions to produce organic acids as well as CO_2_ and H_2_ gasses and ethanol. Microorganisms capable of this process include homofermentative bacteria, which dissimilate glucose solely through the glycolytic pathway to produce lactic acid (e.g., genus *Streptococci* and *Lactobacilli)*, heterofermentative bacteria, which can complete mixed-acid fermentations and produce complex organic acid mixtures (e.g., genus *Escherichia)*^32^, and select fungal species (e.g., genus *Rhizopus)* which are less common but are capable of performing lactic acid fermentation^33^. Of specific interest for use in subsurface colloidal silica applications, glucose fermentation can be performed by a diverse range of microorganisms that are abundant in natural systems, can generate organic acids mixtures with low pKa values needed to enable large pH reductions (e.g., lactic acid pKa = 3.86; acetic acid pKa = 4.76; succinic acid pKa_1_ = 4.21, pKa_2_ = 5.64; formic acid pKa = 3.75)^34^, are capable of tolerating large differences in surrounding environmental conditions, and generate ecologically-inert by-products which do not require post-treatment removal^35^.

A study was performed to examine the potential of glucose fermenting microorganisms to be enriched in natural sands to mediate the gelation of colloidal silica grout suspensions via controlled solution pH reductions and ionic strength increases. A series of batch experiments were first performed to understand the effect of solution pH and sodium chloride (NaCl) additions on the rate of gel formation observed in abiotic colloidal silica suspensions in order to identify targeted final pH ranges needed to design the biomediated process. Following these experiments, three additional series of batch experiments were performed to investigate the ability of glucose fermenting microorganisms to be enriched in natural sands, induce geochemical changes capable of mediating colloidal silica gel formation, and study the effect of initial solution composition, sand material type, and supplied soil-to-solution ratio on pH reduction behaviors. Following the identification of treatment techniques capable of successful enrichment of microbial fermentation activity, a series of complementary batch and soil column experiments were performed to further examine the ability of stimulated glucose fermenting microorganisms to enable control of silica gel formation under conditions more representative of in situ soils, the effectiveness of various monitoring methods to track silica gel formation and microbial activity, and to characterize soil engineering improvements afforded by resulting colloidal silica gels.

## Materials and methods

### Sand materials

Two different poorly graded clean sands, Delta Sand and Concrete Sand, were included in batch and column experiments. Delta Sand is a marine sand consisting of nearly 58% quartz and 42% albite with a D_10_ of 0.19 mm, D_30_ of 0.25 mm, D_60_ of 0.37 mm, and fines content of 1.3%^36^. Concrete Sand is an alluvial sand consisting of nearly 75% quartz and 25% albite with a D_10_ of 0.23 mm, D_30_ of 0.54 mm, D_60_ of 1.54 mm, and fines content of 1.1%. Both sands classify as a poorly graded sand (SP) following ASTM D2487^37^ and have been extensively investigated in previous studies examining biomediated soil improvement processes^36,38–41^. Both soils were expected to be representative other natural clean sands that might require improvement for liquefaction mitigation purposes.

### Treatment solutions

All colloidal silica solutions were prepared using 30% by mass Ludox SM-30 sodium hydroxide-stabilized colloidal silica stock solutions (Grace Chemicals), which contained silica colloids ranging between 7 and 22 nm in diameter and had an initial pH near 10. Solutions included in all experiments contained 6% colloidal silica by mass, prepared by diluting the 30% stock solution with deionized water. Following preparation of a 6% colloidal silica solution, soluble chemical masses were added directly to solutions and solutions were pH-adjusted using either 1 M sodium hydroxide or hydrochloric acid and then filter-sterilized using 0.2-micron filters. Soluble chemicals added to solutions included various concentrations of glucose (anhydrous dextrose, Fisher Scientific), yeast extract (Fisher Bioreagents), and sodium chloride (Fisher Scientific). All solutions apart from those considered in experimental series 1 (described in Section "Experimental series") were pH-adjusted to an initial value of 9.5 intended to prevent abiotic gelation and maintain solution stability in the absence of fermentation activity. All solutions were prepared in equilibrium with atmospheric conditions at 20 °C and were not de-aired prior to use (dissolved oxygen ≈ 9 mg/L).

### Batch experiments

**Figure 1:**
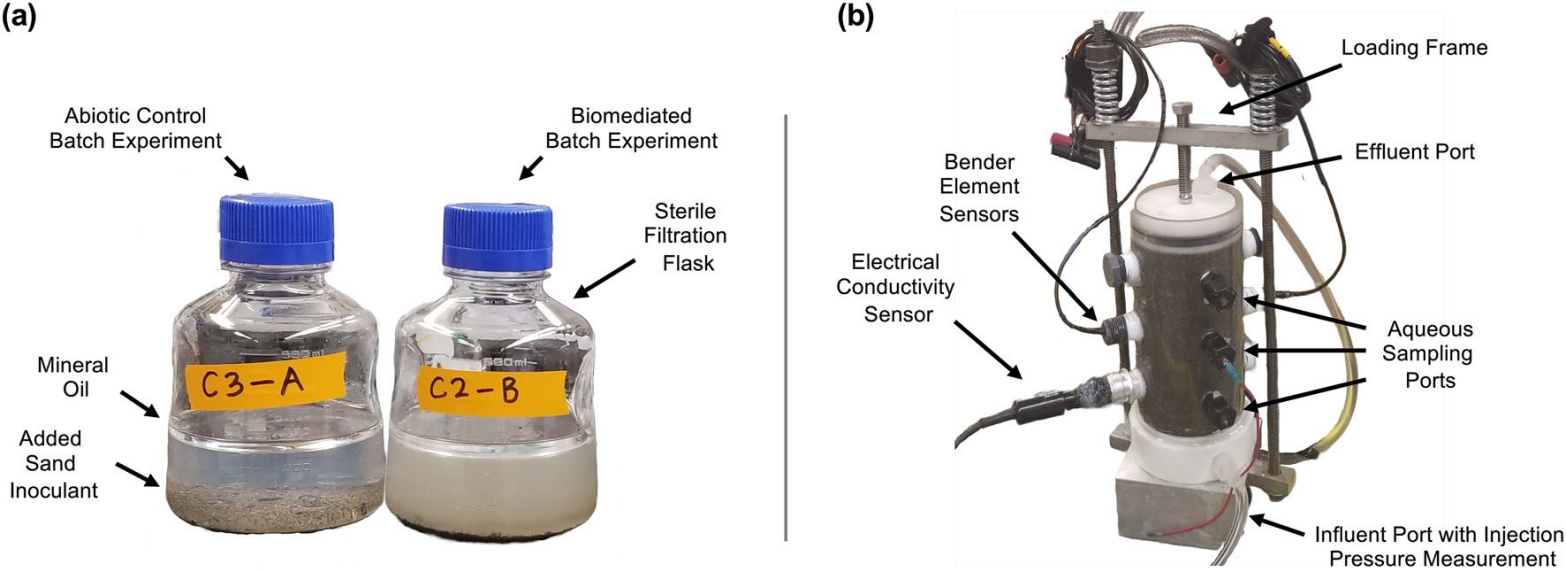
Images of (**a**) select batch experiments including sterile filtration flasks, mineral oil, and added sand inoculants, and (**b**) a single soil column experiment including the column loading frame, bender element sensors, electrical conductivity sensor, aqueous sampling ports, and influent and effluent ports which allowed for connection of pressure transducers for measurements during injections.

All batch experiments included 350 mL volumes of filter-sterilized colloidal silica solutions prepared in sterile 500 mL plastic bottles using vacuum filtration units with 0.2-micron PES filters (Fig. 1a). Following solution filtration, soil masses (when present) were added directly to flasks at varying soil-to-solution ratios ranging from 0.5 to 100 g/L. In order to inhibit oxygen transfer, a 5 mm-thick layer of sterile heavy mineral oil was placed at the surface of all solutions and flasks were sealed using sterile caps. All exposed flask surfaces were flame-sterilized using a portable torch whenever flasks were opened to minimize the potential for contamination. All aqueous samples were obtained using sterile pipettes.

### Soil column experiments

Soil columns specimens were prepared in 15.2 cm high, 7.6 cm inner diameter hollow acrylic cylinders that had PFTE caps on top and bottom for solution exchange and various fittings for bender element sensors, solution sampling ports, and electrical conductivity sensors (Fig. 1b). Three rubber septum sampling ports existed along column heights at three distances from the injection source at the base of columns (5.1, 10.2, 15.2 cm) and were used to obtain solution samples at various times using sterile syringes and needles. Bender element sensor pairs were included at mid-height (10.2 cm from injection source) for all columns to track changes in soil shear wave velocities (V_s_). A single 4-cell electrical conductivity (EC) sensor (range = 1 µS/cm to 200 mS/cm, Fisher Scientific) was also included near the bottom (5.1 cm from injection source) of four select columns to track changes in EC in time resulting from fermentation and gelation. All columns contained Delta Sand and were prepared to an initial relative density of ≈ 40% and had pore volumes (PV) near 250 mL and soil-to-solution ratios near 4500 g/L. Soil materials were retained within columns using porous plastic discs (125–195 μm pore size, Porex Inc.) that were placed at the interface between top and bottom caps and soils to prevent migration of soil materials during injections. Following preparation, all columns were subjected to a vertical total stress of ≈ 100 kPa that was applied using a spring-loaded reaction frame and columns were saturated with deionized water. Following saturation, all columns received a single 8 PV (2 L) stop-flow injection of colloidal silica treatment solutions at a flow rate of 20 mL/min (injection time ≈ 100 min) intended to fully replace residing pore fluids. All columns were treated from the bottom upwards to ensure saturation and minimize possible heterogeneity in injected solutions resulting from solution density differences. Plastic solution reservoirs were connected to column effluent tubes and were filled with ≈ 500 mL of colloidal silica treatment solutions that were exiting columns near the end of injections. Reservoirs remained connected to columns to enable replacement of column pore fluids during sampling and limit oxygen intrusion and desaturation of columns. The total sampled volume for each column was less than 5% of one PV. All monitoring was completed after injections during the subsequent residence period.

### Experimental series

Experiments were performed in five different series intended to investigate the effect of solution composition on stimulated fermentative activity as well as afforded changes in gelation rates and engineering improvements. Experimental series 1 batch experiments first examined the effect of abiotic chemical conditions on solution gelation rates in time as captured by solution viscosity increases. Solutions explored the effect of differences in initial pH values between 4.0 and 10.0 as well as NaCl concentrations between 0 and 10 g/L and were monitored for 120 days. Results from experimental series 1 were used to determine initial pH values which could maintain highly stable suspensions (pH ≈ 9.5) as well as final pH values targeted following fermentation in order to induce colloidal silica gelation (pH ≈ 5.0 to 6.0). Experimental series 2 batch experiments explored the ability of treatment solutions to successfully enrich natural sands for glucose fermenting microorganisms as well as the effect of differences in supplied soil-to-solution ratios on enriched activity. Solutions had an initial pH of 9.5 and included 5 g/L yeast extract (YE), 10 g/L glucose, and different soil-to-solution ratios ranging from 0 to 50 g of soil per L of solution intended to evaluate the abundance of glucose fermenting microorganisms in parent soils. Experiments containing Delta Sand and Concrete Sand were monitored for 10 and 19 days, respectively. Following identification of successful enrichment approaches, experimental series 3 batch experiments further explored the effect of supplied glucose and YE concentrations on pH reduction behaviors. All experiments contained 50 g/L of Delta Sand and included solutions that had an initial pH of 9.5, YE concentrations between 0.1 and 10 g/L, glucose concentrations between 2.5 and 5 g/L, and were monitored for 9 days. Additional batch experiments were performed without added sand to control for potential biological contamination and assess the effect of substrate additions on gelation. Following the identification of solutions capable of obtaining targeted final pH values (≈ 5.0 to 6.0), experimental series 4 batch experiments explored the effect of NaCl additions, which are commonly used as a gelation accelerant. All experiments contained 50 g/L of Delta Sand and solutions that had an initial pH of 9.5 and contained 1 g/L YE, 5 g/L glucose, between 0 and 10 g/L NaCl, and were monitored for 9 days. Table 1 provides a summary of all batch experiments performed in experimental series 1 through 4 including solution compositions and employed monitoring methods.

**Table 1:** Summary of batch experiments from experimental series 1 through 4.

Experimental series	Type	Related figures	Solution composition^a^			Soil additions			Monitoring	
			initial pH	Glucose (g/L)	Yeast extract (g/L)	NaCl (g/L)	Soil type	Soil to solution ratio (g/L)	Viscosity in time	pH in Time
1—Abiotic gelation rate	Batch	Figure 2	4.0	–	–	0	–	–	X	X
1—Abiotic gelation rate	Batch	Figure 2	4.5	–	–	0	–	–	X	X
1—Abiotic gelation rate	Batch	Figure 2	5.0	–	–	0	–	–	X	X
1—Abiotic gelation rate	Batch	Figure 2	5.5	–	–	0	–	–	X	X
1—Abiotic gelation rate	Batch	Figure 2	6.0	–	–	0	–	–	X	X
1—Abiotic gelation rate	Batch	Figure 2	6.5	–	–	0	–	–	X	X
1—Abiotic gelation rate	Batch	Figure 2	7.0	–	–	0	–	–	X	X
1—Abiotic gelation rate	Batch	Figure 2	7.5	–	–	0	–	–	X	X
1—Abiotic gelation rate	Batch	Figure 2	8.0	–	–	0	–	–	X	X
1—Abiotic gelation rate	Batch	Figure 2	8.5	–	–	0	–	–	X	X
1—Abiotic gelation rate	Batch	Figure 2	9.0	–	–	0	–	–	X	X
1—Abiotic gelation rate	Batch	Figure 2	9.5	–	–	0	–	–	X	X
1—Abiotic gelation rate	Batch	Figure 2	10.0	–	–	0	–	–	X	X
1—Abiotic gelation rate	Batch	Figure 2	4.0	–	–	5	–	–	X	X
1—Abiotic gelation rate	Batch	Figure 2	4.5	–	–	5	–	–	X	X
1—Abiotic gelation rate	Batch	Figure 2	5.0	–	–	5	–	–	X	X
1—Abiotic gelation rate	Batch	Figure 2	5.5	–	–	5	–	–	X	X
1—Abiotic gelation rate	Batch	Figure 2	6.0	–	–	5	–	–	X	X
1—Abiotic gelation rate	Batch	Figure 2	6.5	–	–	5	–	–	X	X
1—Abiotic gelation rate	Batch	Figure 2	7.0	–	–	5	–	–	X	X
1—Abiotic gelation rate	Batch	Figure 2	7.5	–	–	5	–	–	X	X
1—Abiotic gelation rate	Batch	Figure 2	8.0	–	–	5	–	–	X	X
1—Abiotic gelation rate	Batch	Figure 2	8.5	–	–	5	–	–	X	X
1—Abiotic gelation rate	Batch	Figure 2	9.0	–	–	5	–	–	X	X
1—Abiotic gelation rate	Batch	Figure 2	9.5	–	–	5	–	–	X	X
1—Abiotic gelation rate	Batch	Figure 2	10.0	–	–	5	–	–	X	X
1—Abiotic gelation rate	Batch	Figure 2	4.0	–	–	10	–	–	X	X
1—Abiotic gelation rate	Batch	Figure 2	4.5	–	–	10	–	–	X	X
1—Abiotic gelation rate	Batch	Figure 2	5.0	–	–	10	–	–	X	X
1—Abiotic gelation rate	Batch	Figure 2	5.5	–	–	10	–	–	X	X
1—Abiotic gelation rate	Batch	Figure 2	6.0	–	–	10	–	–	X	X
1—Abiotic gelation rate	Batch	Figure 2	6.5	–	–	10	–	–	X	X
1—Abiotic gelation rate	Batch	Figure 2	7.0	–	–	10	–	–	X	X
1—Abiotic gelation rate	Batch	Figure 2	7.5	–	–	10	–	–	X	X
1—Abiotic gelation rate	Batch	Figure 2	8.0	-	-	10	-	-	X	X
1—Abiotic gelation rate	Batch	Figure 2	8.5	–	–	10	–	–	X	X
1—Abiotic gelation rate	Batch	Figure 2	9.0	–	–	10	–	–	X	X
1—Abiotic gelation rate	Batch	Figure 2	9.5	–	–	10	–	–	X	X
1—Abiotic gelation rate	Batch	Figure 2	10.0	–	–	10	–	–	X	X
2—Effect of soil additions	Batch	Figure 3	9.5	10	5	0	Concrete	0		X
2—Effect of soil additions	Batch	Figure 3	9.5	10	5	0	Concrete	0.5		X
2—Effect of soil additions	Batch	Figure 3	9.5	10	5	0	Concrete	5		X
2—Effect of soil additions	Batch	Figure 3	9.5	10	5	0	Concrete	50		X
2—Effect of soil additions	Batch	Figure 3	9.5	10	5	0	Delta	0		X
2—Effect of soil additions	Batch	Figures 3 and 6	9.5	10	5	0	Delta	0.5		X
2—Effect of soil additions	Batch	Figures 3 and 6	9.5	10	5	0	Delta	5		X
2—Effect of soil additions	Batch	Figures 3 and 6	9.5	10	5	0	Delta	50		X
3—Effect of glucose & YE Conc	Batch	Figures 4 and 6	9.5	2.5	0.1	0	Delta	50		X
3—Effect of glucose & YE Conc	Batch	Figures 4 and 6	9.5	2.5	1	0	Delta	50		X
3—Effect of glucose & YE Conc	Batch	Figures 4 and 6	9.5	2.5	10	0	Delta	50		X
3—Effect of glucose & YE Conc	Batch	Figures 4 and 6	9.5	3.75	0.1	0	Delta	50		X
3—Effect of glucose & YE Conc	Batch	Figure 4 and 6	9.5	3.75	1	0	Delta	50		X
3—Effect of glucose & YE Conc	Batch	Figures 4 and 6	9.5	3.75	10	0	Delta	50		X
3—Effect of glucose & YE Conc	Batch	Figures 4 and 6	9.5	5	0.1	0	Delta	50		X
3—Effect of glucose & YE Conc	Batch	Figures 4 and 6	9.5	5	1	0	Delta	50		X
3—Effect of glucose & YE Conc	Batch	Figures 4 and 6	9.5	5	10	0	Delta	50		X
3—Effect of glucose & YE Conc	Batch	Figure 4	9.5	5	0.1	0	–	–		X
3—Effect of glucose & YE Conc	Batch	Figure 4	9.5	5	1	0	–	–		X
3—Effect of glucose & YE Conc	Batch	Figures 4	9.5	5	10	0	–	–		X
4—Effect of NaCl Conc	Batch	Figures 5 and 6	9.5	5	1	0	Delta	50		X
4—Effect of NaCl Conc	Batch	Figures 5 and 6	9.5	5	1	1	Delta	50		X
4—Effect of NaCl Conc	Batch	Figures 5 and 6	9.5	5	1	2.5	Delta	50		X
4—Effect of NaCl Conc	Batch	Figures 5 and 6	9.5	5	1	5	Delta	50		X
4—Effect of NaCl Conc	Batch	Figures 5 and 6	9.5	5	1	10	Delta	50		X

*X* =Monitoring method performed.

^a^All experiments contain 6% by mass colloidal silica.

Following insights achieved from earlier batch experiments, experimental series 5 was performed to explore the efficacy of the biomediated process under more field-representative conditions and evaluate the ability of chemical, geophysical, and other characterization processes to track microbial activity, gel formation, and quantify achieved engineering enhancements. Experimental series 5 consisted of complementary soil column and batch experiments that were treated using seven distinct treatment solutions intended to evaluate the effect of solution composition and compare enrichment and gelation behaviors as a function of soil-to-solution ratios. Two different abiotic solutions (Solutions A1 & A2) were employed to evaluate the stability of solutions in the absence of microbial fermentation activity. Both solutions were pH-adjusted to 9.5, contained no added YE or glucose, and included either 0 (Solution A1) or 1 g/L NaCl (Solution A2). Five different solutions (Solutions B1 to B5) were also considered to explore various aspects of the biomediated process including: (i) the effect of supplied YE concentrations in solutions containing either 0.2 g/L (Solution B1), 1 g/L (Solution B2i & B2ii), or 5 g/L (Solution B3) YE with 5 g/L glucose, (ii) the effect of glucose concentrations in solutions containing either 5 g/L (Solution B2i & B2ii) or 10 g/L glucose (Solution B4) with 1 g/L YE, and (iii) the effect of added NaCl in solutions containing either 0 g/L (Solutions B2i & B2ii) or 1 g/L NaCl (Solution B5) with 5 g/L glucose and 1 g/L YE. The repeatability of experiments was assessed through experiments which received identical solution compositions (Solution B2i & B2ii) and were compared to identical experiments also included in experimental series 3 and 4. For each solution, two batch experiments were performed at soil-to-solution ratios of 50 g/L and 100 g/L and a single soil column experiment was performed with a soil-to-solution ratio near 4500 g/L. Batch and soil column experiments performed in experimental series 5 were monitored for up to 6 days and remained untreated for an additional 8 days until post-treatment characterizations of viscosity, unconfined compressive strength, and hydraulic conductivity were completed. Table 2 provides a summary of all soil column and batch experiments performed in experimental series 5 including solution compositions and employed monitoring methods.

**Table 2:** Summary of batch and soil column experiments from experimental series 5.

Experimental series	Type	Related figures	Solution Composition^a^					Soil additions		Monitoring						
			Solution type	Initial pH	Glucose (g/L)	Yeast extract (g/L)	NaCl (g/L)	Soil type	Soil to solution ratio (g/L)	Viscosity in time	pH in time	Glucose in time	EC in time	V_s_ in time	Hyd. cond. (before and after)	UCS (before and after)
5—Column comparison (A1)	Batch	Figures 8, 10, and 13	A1	9.5	–	–	–	Delta	50	X	X	X				
5—Column comparison (A1)	Batch	Figures 8, 10, and 13	A1	9.5	–	–	–	Delta	100	X	X	X				
5—Column comparison (A2)	Batch	Figures 8, 10, and 13	A2	9.5	–	–	1	Delta	50	X	X	X				
5—Column comparison (A2)	Batch	Figures 8, 10, and 13	A2	9.5	–	–	1	Delta	100	X	X	X				
5—Column comparison (B1)	Batch	Figures 8, 10, and 13	B1	9.5	5	0.2	–	Delta	50	X	X	X				
5—Column comparison (B1)	Batch	Figure 8, 10, and 13	B1	9.5	5	0.2	–	Delta	100	X	X	X				
5—Column comparison (B2i)	Batch	Figures 8, 10, and 13	B2i	9.5	5	1	–	Delta	50	X	X	X				
5—Column comparison (B2i)	Batch	Figures 8, 10, and 13	B2i	9.5	5	1	–	Delta	100	X	X	X				
5—Column comparison (B2ii)	Batch	Figures 8, 10, and 13	B2ii	9.5	5	1	–	Delta	50	X	X	X				
5—Column comparison (B2ii)	Batch	Figure 8, 10, and 13	B2ii	9.5	5	1	–	Delta	100	X	X	X				
5—Column comparison (B3)	Batch	Figures 8, 10, and 13	B3	9.5	5	5	–	Delta	50	X	X	X				
5—Column comparison (B3)	Batch	Figures 8, 10, and 13	B3	9.5	5	5	–	Delta	100	X	X	X				
5—Column comparison (B4)	Batch	Figures 8, 10, and 13	B4	9.5	10	1	–	Delta	50	X	X	X				
5—Column comparison (B4)	Batch	Figures 8, 10, and 13	B4	9.5	10	1	–	Delta	100	X	X	X				
5—Column comparison (B5)	Batch	Figures 8, 10, and 13	B5	9.5	5	1	1	Delta	50	X	X	X				
5—Column comparison (B5)	Batch	Figure 8, 10, and 13	B5	9.5	5	1	1	Delta	100	X	X	X				
5—Column Comparison (A1)	Column	Figures 7, 8, 9, 10, 11, 12, and 13	A1	9.5	–	–	–	Delta	4500		X	X	X	X	X	X
5- Column comparison (A2)	Column	Figures 7, 8, 9, 10, 11, 12, and 13	A2	9.5	–	–	–	Delta	4500		X	X	X	X	X	X
5—Column comparison (B1)	Column	Figures 7, 8, 9, 10, 11, 12, and 13	B1	9.5	5	0.2	–	Delta	4500		X	X	X	X	X	X
5—Column comparison (B2i)	Column	Figures 7, 8, 9, 10, 11, 12, and 13	B2i	9.5	5	1	–	Delta	4500		X	X	X	X	X	X
5—Column comparison (B2ii)	Column	Figures 7, 8, 9, 10, 11, 12, and 13	B2ii	9.5	5	1	–	Delta	4500		X	X	X	X	X	X
5- Column comparison (B3)	Column	Figures 7, 8, 9, 10, 11, 12, and 13	B3	9.5	5	5	–	Delta	4500		X	X	X	X	X	X
5—Column Comparison (B4)	Column	Figures 7, 8, 9, 10, 11, 12, and 13	B4	9.5	10	1	–	Delta	4500		X	X	X	X	X	X
5—Column comparison (B5)	Column	Figures 7, 8, 9, 10, 11, 12, and 13	B5	9.5	5	1	1	Delta	4500		X	X	X	X	X	X

*X* = Monitoring method performed.

^a^All experiments contain 6% by mass colloidal silica.

### Aqueous measurements

Solution pH measurements were completed immediately after solution sampling using a semi-micro pH electrode and meter (Orion Versa Star Meter, Thermo Fisher) that was calibrated daily and had ± 0.05 pH unit accuracy. Aqueous glucose concentration measurements were performed exclusively for batch and soil column experiments from experimental series 5 using 125 μL aqueous samples. Solution samples obtained for glucose measurements were stabilized immediately following collection in 1.6 M sodium hydroxide to inhibit microbial activity and dissolve colloidal silica gels, when present. Glucose concentrations were measured using collected samples and a EnzyChromTM Glucose III colorimetric assay kit (BioAssay Systems Inc.) that had a linear detection range between 0.3 and 2.0 mM glucose. Following the addition of the colorimetric reagent, all samples were allowed to equilibrate for 30 min at room temperature and optical densities were measured using a microplate spectrophotometer (Biotek Inc.) at a wavelength of 565 nm.

### Viscosity measurements

For batch experiments in experimental series 1 and 5, solution viscosity changes were assessed using a low-range digital viscometer (DVELV viscometer, Ametek Brookfield). Solution viscosity measurements were obtained only at the beginning and end of the biomediated experiments in order to mitigate the potential for biological contamination of experiments and oxygen intrusion, which could have inhibited fermentation activity. All abiotic non-sterile batch experiments in experimental series 1 had solution viscosities measured once daily.

### Geophysical measurements

Electrical conductivity and V_s_ measurements were performed at various times during soil column experiments to examine the ability of non-destructive geophysical measurements to detect changes in colloidal silica gelation progression and microbial fermentation activity. EC measurements were completed a minimum of three times daily in four select soil columns using embedded sensors. Columns instrumented with EC sensors were designed to evaluate the response of similar solutions applied under abiotic (Solution A1, A2) and biomediated (Solution B2ii, B5) conditions as well as to assess the influence of added NaCl by comparing columns receiving solutions without (Solution A1, B2ii) and with 1 g/L NaCl (Solution A2, B5). V_s_ measurements were completed once daily for all columns using bender element sensors that were excited using a 24 V 100 Hz square wave, with received signals measured and recorded using an oscilloscope at a sampling frequency of 1 MHz following procedures similar to Lee et al. (2022)^42^. Known bender element spacings and measured wave transmission times were used to determine soil V_s_ values at various times after injections. All columns had initial V_s_ values between 90 and 110 m/s.

### Hydraulic conductivity measurements

Fluid pore pressure and flow rate measurements were used to estimate soil hydraulic conductivities both before and after colloidal silica solution treatments. The initial hydraulic conductivity of all soil columns was measured during the 8 PV treatment injection. Final hydraulic conductivities were assessed for all columns 14 days after injections by applying an additional 8 PV of deionized water. During injections, pore pressure measurements were obtained at both column influent and effluent locations, flow rates were measured using solution volumes exiting columns, and hydraulic conductivities were estimated using these measurements, known column geometries, and Darcy’s Law. All pore pressures and flow rates were monitored and allowed to stabilize for at least 10 min prior to determining soil hydraulic conductivities.

### UCS measurements

Soil column specimens were extruded from acrylic cylinders after final hydraulic conductivity testing using a hydraulic extruder. All soil columns that remained intact following extrusion were subjected to unconfined compressive strength (UCS) testing to assess potential increases in tensile strengths afforded by silica grouts. UCS tests were performed using an electro-mechanical loading frame system (GDS Instruments) in accordance with ASTM D2166^43^ using a constant axial strain rate of 1% per minute.

## Results and discussion

### Abiotic gelation rate experiments

**Figure 2:**
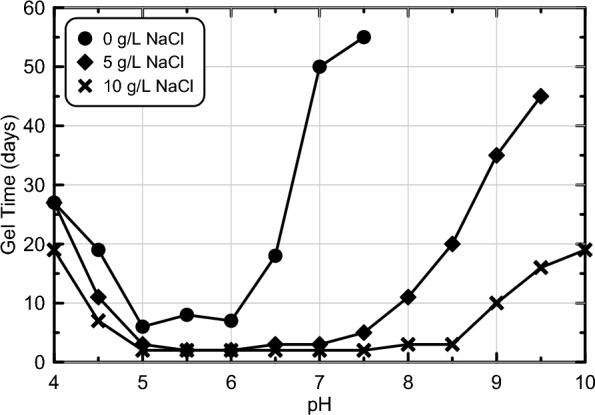
Gel time (time to viscosity > 2000 cP) versus pH for all abiotic colloidal silica batch experiments from experimental series 1 with NaCl concentrations between 0 and 10 g/L.

Figure 2 presents the time required achieve solution viscosities exceeding 2000 cP, referred to as the gel time, versus solution pH for all abiotic colloidal silica batch experiments from experimental series 1. Experiments considered pH values ranging from 4.0 to 10.0 and NaCl concentrations ranging from 0 to 10 g/L, with gel times determined from viscosity measurements in time (shown in Supplemental Fig. S1). Large variations in the rates of viscosity increases were observed with both changes in pH and NaCl concentrations. At the same pH, decreases in gel times were observed with increasing NaCl concentrations. However, for similar NaCl concentrations, solutions prepared to the lowest (pH = 4.0) and highest (pH = 10.0) pH values considered had the longest gel times, with solutions prepared to pH values near 6.0 exhibiting the shortest gel times. As expected, all experiments maintained stable pH values in time following initial mixing thereby confirming that in all experiments gel formation was abiotically induced (Supplemental Figure S2). When no NaCl was supplied, gel times were shortest for pH values between 5.0 and 6.0, wherein most specimens required between 6 and 7 days to reach the gel point. At initial pH values below or above this range, however, gel times increased significantly. For example, at initial pH values greater than 7.5, the gel point was not achieved after 120 days of monitoring. As supplied NaCl concentrations were increased to 5 g/L, the pH range over which gel times were shortest was broadened to include pH values between 5.0 and 7.0 with approximately 2 days required to reach the gel point in most experiments. Again, at pH values below and above this range, however, gel times increased to between 5 and 45 days, with the pH = 10.0 specimen still not able to achieve the gel point within 120 days. At the highest NaCl concentration considered (10 g/L), the pH range over which gel times were shortest was extended to include values between 5.0 and 8.5 with gel times again as low as 2 days. Outside of this pH range, however, gel times were a maximum of only 20 days. This outcome was consistent with other past studies^13^ which have shown that NaCl additions can be used to decrease gel times and broaden optimal pH ranges required for gel formation. In contrast, solutions without NaCl additions remained highly stable once pH values were either less than 4.5 or exceeded 6.5. It was hypothesized that microbial fermentation could exert the greatest control over silica gel formation for alkaline solutions that would otherwise remain highly stable abiotically. Accordingly, solutions with an initial pH of 9.5 and a NaCl concentration of 0 g/L were considered in subsequent experiments and were expected to provide the greatest opportunity for microbial fermentation to control gelation timing through pH reduction rates and final pH reduction magnitudes. In these biomediated experiments, final pH values between 5.0 and 6.0 were targeted following microbial fermentation in order to enable more rapid silica gelation.

### Biomediated batch experiments

#### Effect of sand type and solution-to-soil ratio

**Figure 3:**
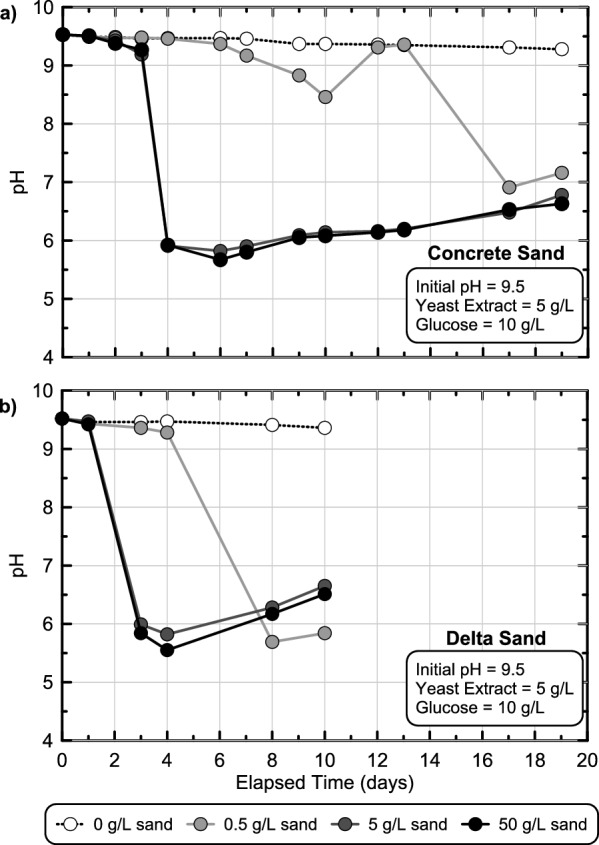
pH measurements versus time obtained from stimulated colloidal silica batch experiments in experimental series 2 containing 0, 0.5, 5, or 50 g/L of (**a**) Concrete Sand or (**b**) Delta Sand. All solutions contained 6% colloidal silica, 5 g/L yeast extract, 10 g/L glucose, and had an initial pH of 9.5.

Figure 3 presents pH measurements in time for batch experiments from experimental series 2 containing Concrete Sand (Fig. 3a) and Delta Sand (Fig. 3b) at varying soil-to-solution ratios. As shown in Fig. 3a, pH reductions from 9.5 to ≈ 7.0 were observed between 4 and 17 days in all Concrete Sand experiments. As soil-to-solution ratios increased, the onset of pH reductions was observed earlier in time suggesting that faster increases in enriched fermentative microbial cell densities could be obtained with increases in added soil masses. This lag time reflected both the time required for fermentative microbial enrichment and cell growth as well as the time required for sufficient glucose fermentation needed to change the surrounding global solution pH, suggesting that increases in added soil masses likely provided a larger initial cell density of fermentative microorganisms which could be enriched, consistent with many other studies demonstrating increases in cell densities with added soil masses^44,45^. For all experiments containing at least 5 g/L soil, pH trends were similar in time and minimum pH values were within the targeted range of 5.0 to 6.0 identified from earlier experiments. When considering trends for Delta Sand, pH reductions from 9.5 to ≈ 7.0 were observed within 2.5–7 days, slightly earlier than Concrete Sand experiments. The faster pH reduction rate observed in Delta Sand in comparison to Concrete Sand may have resulted from either more rapid enrichment of glucose fermenting microorganisms in this soil or a larger initial inoculum of fermentative microorganisms per mass of soil. It was hypothesized that native microorganisms present in Delta Sand may have exhibited greater tolerance to alkaline pH and high ionic strength conditions imposed by the colloidal silica solutions due to the marine origins of this parent soil, thereby increasing the rate of enrichment. Similar to Concrete Sand, pH reduction rates were proportional to added soil masses, with experiments containing at least 5 g/L soil achieving minimum pH values between 5.0 and 6.0. Minimal pH changes were observed in both sterile control experiments which contained no added sand, suggesting that no detectable biological contamination occurred. Although viscosity measurements in time were not obtained for biomediated experiments in order to mitigate the potential for biological contamination and oxygen intrusion, earlier results from Fig. 2 suggested that solution viscosities would be expected to increase to values above 2000 cP approximately 6 to 7 days after achieving pH values between 5.0 and 6.0 and between 7 and 50 days after achieving pH values between 6.0 and 7.0 for experiments without added NaCl.

#### Effect of applied glucose and yeast extract concentration

**Figure 4:**
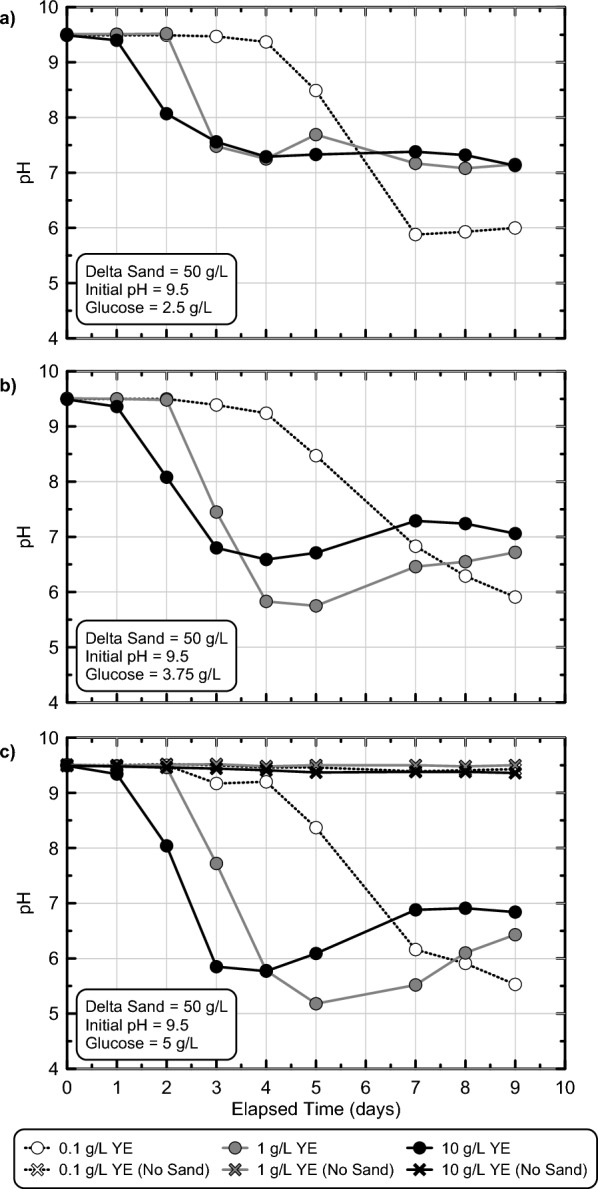
pH measurements versus time obtained from stimulated colloidal silica batch experiments in experimental series 3 containing 0.1, 1, or 10 g/L yeast extract and 2.5, 3.75, or 5 g/L glucose. All solutions contained 6% colloidal silica, 50 g/L Delta Sand, and had an initial pH of 9.5.

Following the identification of successful enrichment techniques, a third set of batch experiments was performed to better understand the role of YE and glucose concentrations towards altering microbial fermentation activity in time. Experimental series 3 considered a narrower range of glucose concentrations intended to achieve final pH values near the targeted range of 5.0–6.0 with minimal material usage and also considered a wide range of YE concentrations that were expected to alter enriched cell densities and therefore control fermentation and pH reduction rates^46^. Figure 4 presents pH measurements in time obtained from batch experiments that received solutions with 2.5–5 g/L glucose, 0.1–10 g/L YE, and 50 g/L Delta Sand. Sterile control experiments were also performed which contained no added sand. As shown, when 2.5 g/L glucose was present, pH reductions from 9.5 to ≈ 8.0 were observed in all experiments within 2 to 5.5 days (Fig. 4a). Detectable pH reductions also occurred earlier in time and at a faster rate for specimens with higher YE concentrations reflective of increases in enriched fermentation activity. Although similar minimum pH values between 7.0 and 7.5 were observed in the higher YE experiments, in the 0.1 g/L YE experiment, interestingly, a lower minimum pH near ≈ 6.0 was obtained, possibly reflective of more selective enrichment and/or difference in solution buffering. When considering similar trends for all experiments containing 3.75 g/L glucose (Fig. 4b), again pH reductions from 9.5 to ≈ 8.0 were observed in all experiments between 2 and 5.5 days and pH reduction activity was proportional to supplied YE. Lower minimum pH values were observed, however, to values between 5.5 and 6.5, which were near the targeted pH range. Finally, in experiments receiving 5 g/L glucose (Fig. 4c), similar pH reductions from 9.5 to ≈ 8.0 were observed within 2–6 days and minimum pH values between 5.0 and 6.0 were achieved within 3–9 days. All sterile control experiments exhibited minimal pH changes regardless of YE concentration, again reflective of continued sterility in time. Collectively, these results suggested that 5 g/L glucose provided sufficient fermentable carbohydrates needed to achieve the targeted pH values with YE providing a means by which fermentation and gelation rates could be controlled. Differences in the time required to achieve initial pH reductions from 9.5 to 8.5 and 9.5 to 7.5 were also characterized (Supplemental Fig. S3), which were expected to best reflect differences in fermentative microbial densities due to the limited dependence of fermentation rates on glucose concentrations near the start of reactions at high glucose concentrations, in accordance with Michaelis–Menten kinetics. As expected, initial pH reduction rates were strongly correlated with changes in supplied YE but were largely independent of differences in supplied glucose. Furthermore, while large pH reduction rate differences could be observed as YE was varied between 0.1 and 1 g/L, when YE was further increased to 10 g/L, more minimal increases in rates were observed, suggesting that the growth and enrichment of fermentative microorganisms was likely limited by other factors such as cellular waste generation and/or trace nutrient limitations at high YE concentrations.

#### Effect of applied NaCl concentration

**Figure 5:**
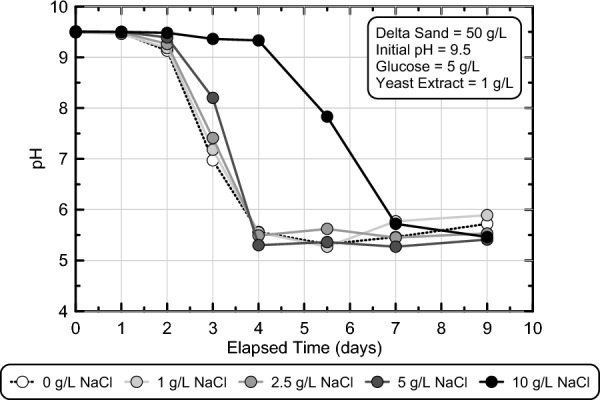
pH measurements versus time from stimulated colloidal silica batch experiments in experimental series 4 containing 0, 1, 2.5, 5, and 10 g/L NaCl. All solutions contained 6% colloidal silica, 50 g/L Delta Sand, 5 g/L glucose, 1 g/L yeast extract, and had an initial pH of 9.5.

Although earlier abiotic experiments demonstrated that NaCl additions could be used to broaden the range of pH values required to expedite the gelation process, it remained unclear what effect added NaCl might have on the enrichment of fermenting microorganisms when included in suspensions to further accelerate gelation. A series of batch experiments were conducted in experimental series 4 to assess the effect of NaCl additions on enriched fermentation activity. Although higher NaCl concentrations were expected to slow the enrichment process and microbial fermentation activity through increases in osmotic stress and potential interferences with cellular transport processes, past studies have shown that *Escherichia coli*, a model mixed acid fermenting microorganism, exhibited both increases^47^ and decreases^48^ in fermentation activity depending on surrounding geochemical conditions. Figure 5 presents pH measurements in time for batch experiments containing solutions with 0 to 10 g/L NaCl, 50 g/L Delta Sand, 5 g/L glucose, and 1 g/L YE, with an initial pH of 9.5. As shown, minimal differences in pH reduction behaviors were observed when NaCl concentrations were less than or equal to 5 g/L with all experiments achieving pH values between 5.0 and 6.0 within 4 days. In the 10 g/L NaCl experiment, however, significant inhibition of fermentation activity was observed with a delay in the onset of pH reductions of about 2 days and a delay in the presence of pH values within the target range near 3 days suggesting that enriched fermentation activity may be inhibited at high salt concentrations. Again, earlier results from Fig. 2 suggested that solution viscosities would be expected to increase to values above 2000 cP approximately 6 to 7 days, 2–3 days, and near 2 days after achieving pH values between 5.0 and 6.0 in experiments containing 0, 5, and 10 g/L NaCl, respectively.

#### Effect of applied glucose concentrations on pH reduction magnitudes

**Figure 6:**
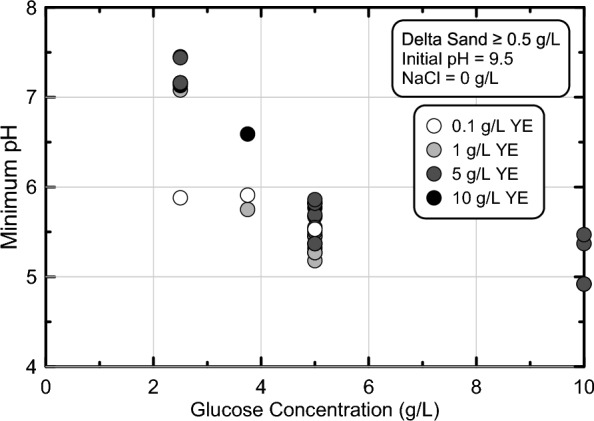
Minimum pH versus initial glucose concentrations for stimulated colloidal silica batch experiments from experimental series 2 through 4. All solutions contained 6% colloidal silica, greater than 0.5 g/L Delta Sand, 0 g/L NaCl, between 2.5 and 10 g/L glucose, between 0.1 and 10 g/L YE, and had an initial pH of 9.5.

In order to further understand the effect of applied glucose concentrations on minimum pH values realized in batch experiments, minimum pH values were plotted versus supplied glucose concentrations for all experiments from experimental series 2 through 4 containing between 2.5 and 10 g/L glucose, between 0.1 and 10 g/L YE, 0 g/L NaCl, and Delta Sand soil-to-solution ratios greater than 0.5 g/L (Fig. 6). As shown, increases in supplied glucose concentrations resulted in near linear reductions in minimum pH values when supplied glucose concentrations were varied from 0 to ≈ 5 g/L. As glucose concentrations further increased, however, more minimal decreases in minimum pH values were observed likely due to the increase buffering of solutions by produced organic acids. When considering the targeted minimum pH range of 5.0 to 6.0, all experiments receiving solutions with at least 5 g/L glucose achieved minimum pH values within the targeted range, despite differences in fermentation rates.

### Upscaling of the biomediated process through soil column experiments

**Figure 7:**
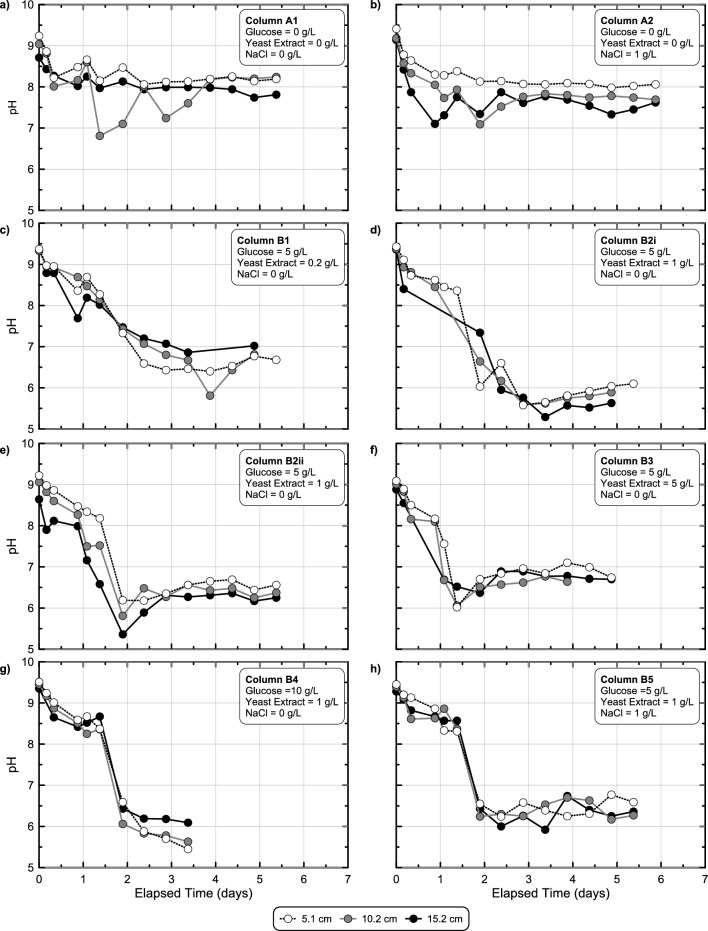
pH measurements versus time from stimulated colloidal silica soil column experiments from experimental series 5 including experiments receiving (**a, b**) two different abiotic and (**c, d, e, f, g, h**) six different biomediated solutions. All solutions contained 6% colloidal silica and varying glucose, YE, and NaCl concentrations with an initial pH of 9.5. Measurements were obtained at three sampling port locations that were 5.1 cm, 10.2 cm, and 15.2 cm from the injection source.

Although the previous batch experiments investigated the effect of treatment solution design on enriched fermentation activity, it remained unclear how the enrichment process might differ under conditions more representative of subsurface soils as well as how the biomediated process could be effectively monitored in situ. A series of complementary batch and soil column experiments were performed in experimental series 5 to investigate the ability of previously identified treatment techniques to be upscaled to more representative soil volumes, the ability of chemical and geophysical monitoring methods to track process progression, and to quantify post-treatment engineering improvements. Figure 7 presents pH measurements versus time after injection measured at three different locations at varying distances from the injection source for all soil column experiments which received a single injection of one of seven different solutions, including two different abiotic solutions (Solutions A1 & A2) and five solutions designed to induce biomediated gelation (Solutions B1 to B5). As shown, in both abiotic columns (Fig. 7a,b) initial pH values were near 9.5, but decreased to between 8.0 and 7.5 within the first day due to equilibration of solutions with existing soil minerals. Although observed pH trends were similar between sampling locations, pH values remained slightly elevated near the injection source, which received more concentrated solutions. When examining trends for biomediated columns (Fig. 7c–h), more minimal differences were observed between sampling port locations suggesting that minimal differences in enriched microbial activity existed spatially within columns. Irrespective of differences in treatment solution compositions between biomediated columns, pH values reduced from initial values near 9.5 to values below 7.0 within 2 days with minimum pH values between 7.0 and 5.0, near and within the target pH range.

**Figure 8:**
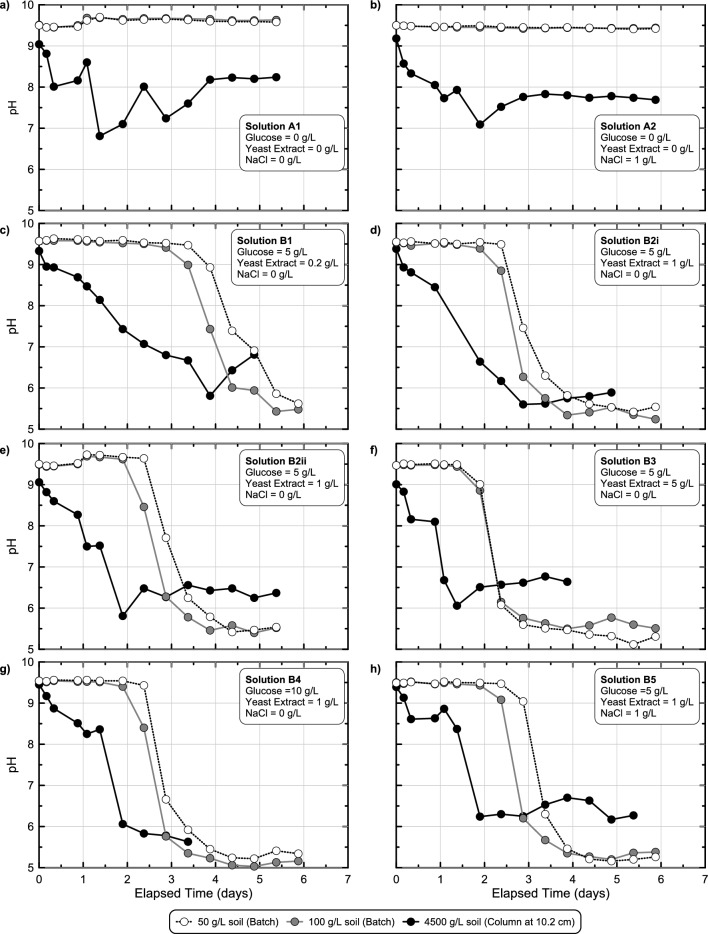
Comparison of pH measurements versus time obtained from stimulated colloidal silica batch and soil column experiments from experimental series 5 including experiments receiving (**a, b**) two different abiotic and (**c, d, e, f, g, h**) six different biomediated solutions. All solutions contained 6% colloidal silica and varying glucose, YE, and NaCl concentrations with an initial pH of 9.5 and considered changes in pH responses with differences in soil-to-solution ratios.

Since all soil column sampling locations had similar pH responses, measurements from only the mid-height sampling location were considered in further comparisons. Figure 8 presents pH measurements in time from all mid-height sampling locations from soil columns as well as corresponding batch experiments receiving identical solutions but with significantly lower soil-to-solution ratios. As shown, for abiotic solutions (Fig. 8a,b) no detectable pH changes were observed in batch experiments, however, appreciable pH reductions were observed in soil columns, attributable to the higher soil-to-solution ratios present in these experiments. When comparing responses observed for biomediated solutions (Fig. 8c–h), faster pH reductions were observed in soil columns when compared to batch experiments, reflective of the higher soil-to-solution ratios that provided larger initial microbial densities, an outcome consistent with trends observed for other biomediated processes^49^. In all batch experiments, minimum pH values within the targeted pH range of 5.0–6.0 were achieved between 2.5 and 5.5 days, consistent with the earlier experiments. Batch experiments containing 100 g/L Delta Sand achieved faster pH reductions than 50 g/L experiments for the same solution, although differences in pH trends had no more than a 1-day offset. Although pH reduction rates were faster initially in soil columns when compared to batch experiments, interestingly, minimum pH values were detectably higher in columns and ranged between 5.5 and 6.5. Lastly, experiments receiving identical solutions (Solutions 2Bi & 2Bii) demonstrated similar pH reduction behaviors in time for both batch experiments and soil columns. For the 50 g/L Delta Sand batch experiments, responses were also compared to identical batch experiments performed in experimental series 3 and 4 with near identical pH behaviors observed in all experiments suggesting that conditions between experimental series were similar and experiments were repeatable (Supplemental Fig. S4). When comparing aqueous glucose concentration trends in time between batch and column experiments similar trends were observed (Supplemental Fig. S5). In all experiments, initial glucose concentrations varied between 5 and 10 g/L with the onset of glucose degradation occurring within 1 to 3 days and increases in glucose degradation rates observed with increases in soil-to-solution ratios. Although near full degradation of the supplied glucose was detected in all 5 g/L glucose soil columns, in the 10 g/L glucose column experiments and select batch experiments, solutions gelled prior to achieving full glucose degradation and aqueous samples could not be physically obtained to capture the end of the fermentation reactions.

**Figure 9:**
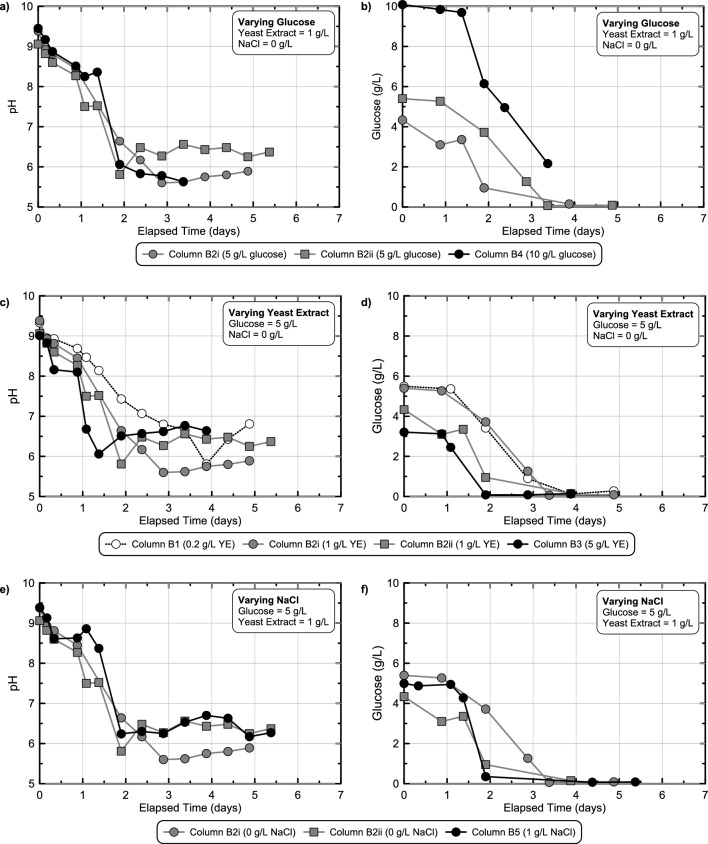
Comparison of pH and glucose measurements in time obtained from mid-height sampling port locations for stimulated colloidal silica soil column experiments from experimental series 5 with differing (**a, b**) glucose, (**c, d**) YE, and (**e, f**) NaCl concentrations. All solutions contained 6% colloidal silica and had an initial pH of 9.5.

In order to understand the effect of various changes in treatment solution formulations on fermentation activity observed in soil column experiments, pH and glucose concentration measurements in time obtained from mid-height sampling ports were compared between select columns (Fig. 9). pH and glucose concentration trends were first compared between soil columns which received similar solutions (1 g/L YE, no added NaCl) but varying initial glucose concentrations (5 g/L or 10 g/L) (Fig. 9a,b). As shown, pH trends were similar between columns in time with minimum pH values between 5.5 and 6.5 observed in all columns, despite significantly greater glucose degradation in the 10 g/L glucose column, an outcome consistent with earlier batch experiments. However, when differences in supplied YE concentrations were considered (Fig. 9c,d) large differences in pH reduction and glucose degradation rates were observed. While the 5 g/L YE column achieved a pH near 6.5 after 1 day, the 1 g/L YE columns achieved this same reduction between 1.5 and 2 days, with the 0.1 g/L YE column requiring nearly 3.5 days. Glucose concentration measurements reflected similar differences in fermentation rates with near full degradation of the supplied 5 g/L glucose observed between 2 and 4 days in all columns. Collectively these results further confirmed the ability of supplied YE concentrations to control enriched fermentative cell densities and reaction rates under conditions more representative of subsurface soils. When considering differences in supplied NaCl (Fig. 9e,f), no significant effects on pH and glucose concentrations trends in time were observed and were consistent with results from earlier batch experiments showing limited inhibition below 5 g/L NaCl. Lastly, columns receiving the same solutions (Solution 2Bi and 2Bii) again demonstrated similar pH and glucose reduction behaviors in time, although with greater variability than batch experiments.

**Figure 10:**
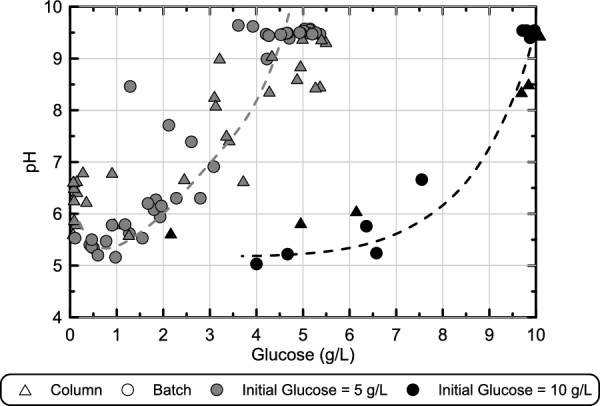
Comparison of corresponding pH and glucose measurements obtained at similar times during reactions for stimulated colloidal silica batch and soil column experiments from experimental series 5 receiving solutions with either 5 or 10 g/L glucose. All solutions contained 6% colloidal silica, had an initial pH of 9.5, and contained varying YE and NaCl concentrations.

In order to better understand relationships between glucose consumption and afforded pH reductions, corresponding measurements of pH and glucose obtained at similar times during reactions (shown in Fig. 9) were compared for all biomediated batch and soil column experiments from experimental series 5 (Fig. 10). As shown, values from all experiments clustered on the basis of initial glucose concentrations with differences in YE and NaCl concentrations having minimal effects on the observed trends (Supplemental Fig. S6). Initial pH values started near 9.5 in all experiments prior to any detectable glucose fermentation with pH values near 6.0 and 5.5 obtained after degradation of 2 g/L and 4 g/L glucose, respectively, regardless of initial glucose concentration. In the 10 g/L glucose experiments, samples with less than 4 g/L glucose could not be obtained due to the gelation of solutions which prevented further sampling. However, for glucose concentrations of less than 6 g/L, pH values appeared to remain largely constant near 5.5 due to solution buffering. Although similar trends were observed between batch and soil column experiments, slightly elevated pH values were observed in soil columns for the same magnitude of glucose degradation. However, the consistency in the broader trends observed in both batch and soil column experiments, demonstrated the utility of batch experiments towards enabling effective treatment solution design and the assessment of site-specific conditions, which may impose solution chemistries that differ from those examined in this study.

**Figure 11:**
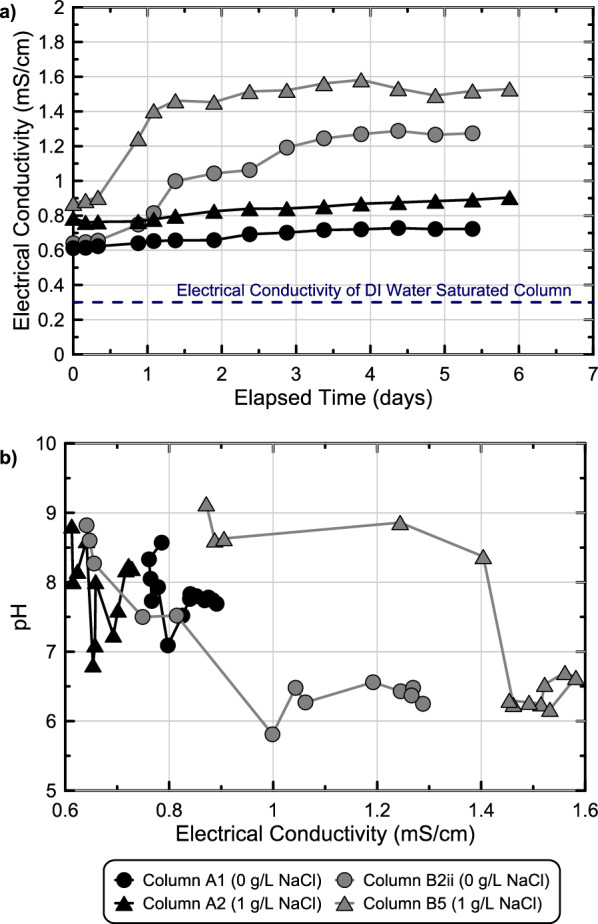
Measurements of (**a**) soil electrical conductivities in time obtained from select soil column experiments from experimental series 5 and (**b**) comparisons of corresponding solution pH and soil electrical conductivity measurements for similar columns.

Figure 11 presents soil electrical conductivity measurements obtained from select soil columns versus time as well as corresponding pH measurements. As shown, immediately after columns were saturated with deionized water, measured EC values were near 0.3 mS/cm in all columns (Fig. 11a). Following colloidal silica solution injections, however, EC values increased significantly to near 0.75 mS/cm and 0.6 mS/cm for columns receiving solutions with and without 1 g/L NaCl, respectively. During the 6-day monitoring period, both abiotic columns maintained relatively stable EC values with only gradual increases of ≈ 0.15 mS/cm over time. In contrast, large increases in soil column EC values were observed after ≈ 1 day in the biomediated column with 1 g/L NaCl (Column B5) and after ≈ 1.5 days when added NaCl was not present (Column B2ii). After 1.5–3.5 days, near stable EC values were observed in both biomediated columns with overall EC increases near 0.7 mS/cm from post-injection values. It was unclear if EC increases were related to solution ionic strength increases resulting from glucose fermentation or other EC changes related to the formation of bonds between silica colloids and the potential release of sorbed electrolytes during gelation^50,51^. In order to assess the effect of fermentation activity on EC increases, corresponding pH and EC values were compared (Fig. 11b). As shown for both abiotic columns, pH values gradually decreased from ≈ 9.0 to between 8.0 and 7.0 over time, while only minimal increases in EC values were observed. In the biomediated column without added NaCl (Column B2ii), pH reductions corresponded with gradual increases in EC. In the biomediated column with 1 g/L NaCl (Column B5), however, a large increase in EC was observed while the pH remained constant near 8.5 and minimal EC increases were observed as the pH changed substantially from 8.5 to 6.0. Although pH decreases and EC increases were not consistently correlated for the two solutions considered, EC increases observed in both biomediated experiments may have resulted from either the gelation of solutions and related changes in stabilizing electrolyte concentrations or solution ionic strength increases afforded by glucose fermentation. Moving forward, EC, glucose, and pH measurements may provide new and more effective methods by which the in situ progression of microbial activity and colloidal silica gelation can be monitored.

**Figure 12:**
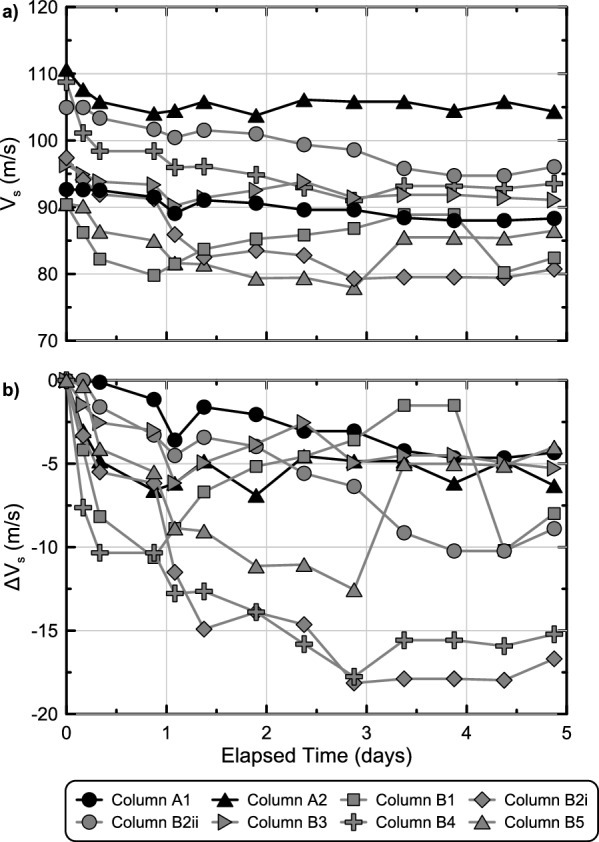
Measurements of (**a**) soil shear wave velocities (V_s_) and (**b**) soil shear wave velocity changes (ΔV_s_) in time obtained for all soil column experiments from experimental series 5 using bender element sensors located at mid-height locations.

Figure 12 presents soil shear wave velocities (V_s_) and shear wave velocity differences (ΔV_s_) in time for all soil columns measured at column mid-heights. Prior to injections, all columns had initial V_s_ values between 90 and 110 m/s with small reductions in V_s_ values observed in the 5 days following injections (Fig. 12a). Although it was unclear why soil V_s_ values decreased over time in both abiotic and biomediated columns, it was hypothesized that such decreases may have been related to the relaxation of applied vertical stresses from top caps over time as well as the swelling of supplied colloidal silica solutions during gelation. ΔV_s_ values further indicated that in both abiotic columns, ΔV_s_ reductions in time near 5 m/s were observed, however, generally larger ΔV_s_ reductions up to 18 m/s were observed in biomediated columns wherein the swelling of colloidal silica solutions may have decreased interparticle contact stresses despite also imparting a small tensile strength (Fig. 12b). This outcome was consistent with observations from other past studies involving sands treated with colloidal silica solutions wherein similar ΔV_s_ reductions were attributed to reductions in interparticle contact stresses following gel formation^6^.

**Figure 13:**
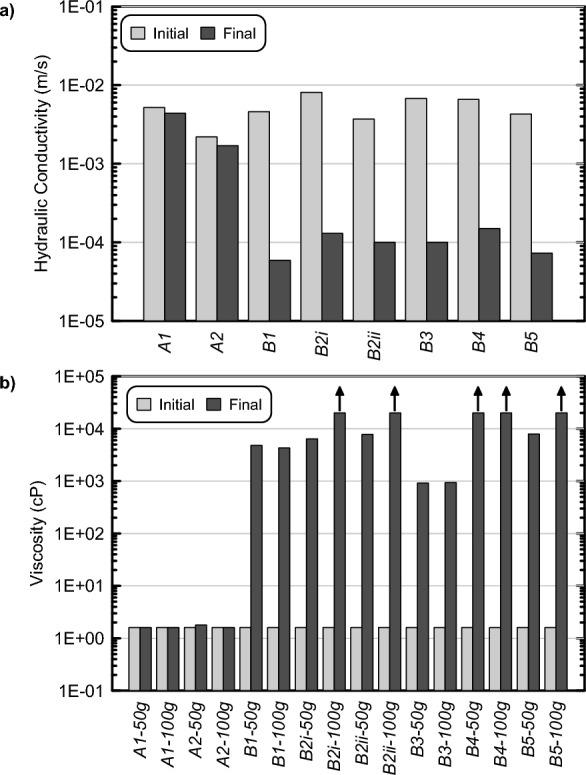
Comparison of initial and final (**a**) hydraulic conductivities from soil column experiments and (**b**) solution viscosities from batch experiments with 50 and 100 g of Delta Sand from experimental series 5. Upward arrows indicate solution viscosities exceeding 20,000 cP which could not be determined.

Following all non-destructive monitoring activities, changes in soil hydraulic conductivities and unconfined compressive strengths (UCS) for all soil column and changes in solution viscosities for all batch experiments were evaluated 14 days after initial injections. Figure 13 presents a comparison of both initial and final hydraulic conductivities for soil column experiments and initial and final viscosities for batch experiments with Supplemental Table S1 providing a summary of all post-treatment characterizations including measured unconfined compressive strengths (UCS) for soil columns. When comparing initial and post-treatment measurements, soil column hydraulic conductivities decreased between 1.5 and 2 orders of magnitude in all biomediated columns which experienced colloidal silica gelation. These hydraulic conductivity reductions were consistent with those observed in other past studies involving sands treated with conventional colloidal silica grouts^52^. In contrast, in both abiotic columns, final soil hydraulic conductivities were only slightly smaller than the initial values, reflective of incomplete gelation. Unconfined compressive strengths (UCS) were also obtained for all four biomediated columns that did not have embedded EC sensors and varied between ≈ 12 and 34 kPa, while both abiotic columns lacked sufficient tensile strength needed to perform UCS measurements. Gallagher and Mitchell (2002)^3^ observed similar UCS values near 35 kPa for 5% colloidal silica treated specimens and demonstrated that such improvements were capable of achieving large increases in soil liquefaction resistances. For example, while an untreated loose sand specimen achieved 5% double amplitude shear strain after 20 cycles of loading at an applied cyclic stress ratio (CSR = τ/σ'_v initial_) of 0.23, a 5% colloidal silica treated specimen required 100 cycles of loading at the same CSR to obtain these same shear strains. When examining changes in solution viscosities for batch experiments, all biomediated batch experiments experienced large viscosity increases from initial values near 1.6 cP to values between 920 cP and exceeding 20,000 cP. In contrast, all abiotic experiments maintained final viscosity values similar to initial values, reflective of the absence of gelation. Collectively these post-treatment characterizations confirmed the ability of microbial fermentation to mediate colloidal silica gelation with final engineering improvements consistent with those afforded by conventional abiotic colloidal silica grouts.

## Conclusions

The potential of enriched fermentative microorganisms to mediate the gelation of colloidal silica grouts via controlled solution pH reductions and ionic strength increases was explored through a series of batch and soil column experiments. Experiments demonstrated microbial fermentation could be used to successfully mediate colloidal silica grouting with the effect of treatment solution composition and effects on soil mechanical properties also examined. From the results of this study, the following conclusions can be made:

- Abiotic batch experiments determined that when 6% colloidal silica solutions contained no added NaCl, gelation occurred most rapidly when pH values were between 5.0 and 6.0. However, when solutions were adjusted to more alkaline pH values near 9.5, solutions remained highly stable and exhibited minimal viscosity changes even after 120 days. Highly stable alkaline (pH = 9.5) suspensions were used in all subsequent biomediated experiments wherein the ability of microbial fermentation activity to reduce pH values and mediate gelation was explored.
- Developed solutions were shown to successfully enrich for microbial glucose fermentation in two different natural sands with increases in soil-to-solution ratios resulting in faster pH reduction rates due to larger initial cell inoculants. Successful enrichment in experiments receiving low soil-to-solution ratios (0.5 g/L), further suggested that fermentative microorganisms capable of facilitating the biomediated process are likely ubiquitous in natural sands.
- Variations in supplied glucose concentrations were shown to control the magnitude of pH reductions observed following injections with variations in supplied yeast extract concentrations altering the onset and rate of pH reductions in time.
- When colloidal silica solutions included variations in supplied NaCl, comparable microbial fermentation activities were observed when NaCl concentrations were at or below 5 g/L suggesting that the process can be used in combination with abiotic gelation accelerants.
- Consistency in the trends observed between batch and soil column experiments as a function of treatment solution composition demonstrated the utility of batch experiments towards enabling effective treatment solution design and the preliminary assessment of site-specific conditions.
- Biomediated batch experiments exhibited large increases in solution viscosities following microbial fermentation (> 2000 cP) reflective of successful gelation, while similar abiotic specimens exhibited no detectable viscosity changes after 14 days.
- In soil column experiments receiving biomediated solutions, electrical conductivities increased significantly in time as colloidal silica grouts gelled suggesting that such measurements may be capable of monitoring the biomediated process. Moving forward, a combination of electrical conductivity, pH, and glucose concentration measurements may provide effective process monitoring techniques for colloidal silica grouting for which few methods currently exist.
- Biomediated soil columns achieved hydraulic conductivity reductions up to 2 orders of magnitude and unconfined compressive strengths (UCS) up to 34 kPa following the biomediated silica gelation process. Measured improvements were consistent with observations from other studies^3^ involving specimens treated using conventional abiotic colloidal silica grouts and suggest that the process may be useful for liquefaction mitigation and seepage control applications.

The biomediated soil improvement technology proposed and developed in this study may provide a method to improve problematic soils with reductions in environmental impacts while addressing key limitations of existing biomediated processes. This includes the ability to improve soils under more acidic conditions, realize large hydraulic conductivity reductions, and eliminate ecologically challenging process by-products. Although these results are promising, future work remains needed to (1) further characterize the mechanical behaviors of soils improved using biomediated colloidal silica grouts, (2) explore the potential of other process verification and monitoring techniques to assess microbial fermentation activity and gelation, (3) investigate the effect of gas phases that may be produced during microbial fermentation on pore-fluid compressibility and undrained soil shearing behaviors, (4) examine the effect of aqueous species that may be present in natural soils and groundwater on process efficacy, (5) further examine temporal changes in solution viscosities for biomediated experiments while maintaining sterility and anoxic conditions, and (6) further quantify process advantages when compared to conventional colloidal silica grouting techniques.

### Supplementary Information

Supplementary Information.

## Data Availability

The datasets generated during and analyzed during the current study are available from the corresponding author on reasonable request.
