# Supplementary material for: Biomediated control of colloidal silica grouting using microbial fermentation
Source: Sci Rep. 2023 Aug 30;13:14184. doi: 10.1038/s41598-023-41402-z (PMC10468516; doi:10.1038/s41598-023-41402-z)
Supplement: Supplementary file 1 — Supplementary Information. [file 41598_2023_41402_MOESM1_ESM.docx]

**Supplementary Material**

**Title:** Biomediated Control of Colloidal Silica Grouting Using Microbial Fermentation

**Authors:** Michael G. Gomez^1^, Samantha T. Muchongwe^2^, Charles M.R. Graddy^3^

^1^Assistant Professor, Department of Civil and Environmental Engineering, University of Washington, 132G More Hall, Seattle, WA, 98195, email: mggomez@uw.edu, ORCID: 0000-0002-4464-544 (Corresponding Author)

^2^M.S. Student, Department of Civil and Environmental Engineering, University of Washington, Seattle, WA, 98195, email: sam.muchongwe@gmail.com, ORCID: 0000-0003-2515-157X

^3^Ph.D. Student, Department of Microbiology and Molecular Genetics, University of California, Davis, Davis, CA 95616, email: cmgraddy@ucdavis.edu, ORCID: 0000-0002-4432-9823

**Supplemental Figure S1.** Measurements of suspension viscosities in time for select batch experiments from experimental series 1 prepared to initial pH values of 4, 6, 8, and 10 with NaCl concentrations of 0, 5, or 10 g/L. Symbol shape denotes NaCl concentration and symbol color denotes initial pH values.

**Supplemental Figure S2.** pH measurements in time for all batch experiments from experimental series 1.

******

**Supplemental Figure S3.** Time to pH values of (**a)** 8.5 and **(b)** 7.5 for stimulated colloidal silica batch experiments from experimental series 3 containing 0.1, 1, or 10 g/L yeast extract and 2.5, 3.75, or 5 g/L glucose. All solutions contained 6% colloidal silica, 50 g/L Delta Sand, and had an initial pH of 9.5.

**Supplemental Figure S4.** Comparison of pH reduction behaviors from identical batch experiments from experimental series 3, 4, and 5 (Solution 2Bi & 2Bii) containing 50 g/L Delta Sand, 1 g/L yeast extract, 5 g/L glucose, 0 g/L NaCl, and 6% colloidal silica, with an initial pH of 9.5.

******

**Supplemental Figure S5.** Comparison of glucose measurements versus time from stimulated colloidal silica columns and batch experiments receiving different biomediated solutions (Solution B1 to B5). All solutions contained 6% colloidal silica and varying glucose, YE, and NaCl concentrations with an initial pH of 9.5 and considered changes in pH responses with differences in soil-to-solution ratios.

**Supplemental Figure S6.** Comparison of corresponding pH and glucose measurements obtained at similar times during reactions for stimulated colloidal silica batch and soil column experiments from experimental series 5. All solutions contained 6% colloidal silica, had an initial pH of 9.5, and contained varying YE, glucose, and NaCl concentrations. As shown, differences in **(a)** NaCl and **(b)** YE concentrations had minimal effects on relationships with initial glucose concentrations (5 or 10 g/L) being the primary factor governing trends, as shown in Figure 10.

**Supplemental Table S1.** Summary of Post-treatment Properties for Batch and Soil Column Experiments from Experimental Series 5

|  |  | **Solution Composition^4^** | | | | | **Soil Additions** | | **Post-treatment Properties** | | | | | | |
| --- | --- | --- | --- | --- | --- | --- | --- | --- | --- | --- | --- | --- | --- | --- | --- |
| **Experimental Series** | **Type** | **Solution Type** | **Initial pH** | **Glucose (g/L)** | **Yeast Extract (g/L)** | **NaCl (g/L)** | **Soil Type** | **Soil to Solution Ratio (g/L)** | **Initial Viscosity (cP)** | **Final x(cP)** | **Final to Initial Viscosity Ratio (%)** | **Initial Hydraulic Conductivity (m/s)** | **Final Hydraulic Conductivity (m/s)** | **Final to Initial Hydraulic Conductivity Ratio (%)** | **UCS (kPa)** |
| 5- Column Comparison (A1) | Batch | A1 | 9.5 | - | - | - | Delta | 50 | 1.6 | 1.6 | 1.0 |  |  |  |  |
| 5- Column Comparison (A1) | Batch | A1 | 9.5 | - | - | - | Delta | 100 | 1.6 | 1.6 | 1.0 |  |  |  |  |
| 5- Column Comparison (A2) | Batch | A2 | 9.5 | - | - | 1 | Delta | 50 | 1.6 | 1.8 | 1.1 |  |  |  |  |
| 5- Column Comparison (A2) | Batch | A2 | 9.5 | - | - | 1 | Delta | 100 | 1.6 | 1.6 | 1.0 |  |  |  |  |
| 5- Column Comparison (B1) | Batch | B1 | 9.5 | 5 | 0.2 | - | Delta | 50 | 1.6 | 4800 | 3000 |  |  |  |  |
| 5- Column Comparison (B1) | Batch | B1 | 9.5 | 5 | 0.2 | - | Delta | 100 | 1.6 | 4300 | 2688 |  |  |  |  |
| 5- Column Comparison (B2i) | Batch | B2i | 9.5 | 5 | 1 | - | Delta | 50 | 1.6 | 6400 | 4000 |  |  |  |  |
| 5- Column Comparison (B2i) | Batch | B2i | 9.5 | 5 | 1 | - | Delta | 100 | 1.6 | > 20000^1^ | > 12500 |  |  |  |  |
| 5- Column Comparison (B2ii) | Batch | B2ii | 9.5 | 5 | 1 | - | Delta | 50 | 1.6 | 7800 | 4875 |  |  |  |  |
| 5- Column Comparison (B2ii) | Batch | B2ii | 9.5 | 5 | 1 | - | Delta | 100 | 1.6 | > 20000^1^ | > 12500 |  |  |  |  |
| 5- Column Comparison (B3) | Batch | B3 | 9.5 | 5 | 5 | - | Delta | 50 | 1.6 | 920 | 575 |  |  |  |  |
| 5- Column Comparison (B3) | Batch | B3 | 9.5 | 5 | 5 | - | Delta | 100 | 1.6 | 940 | 588 |  |  |  |  |
| 5- Column Comparison (B4) | Batch | B4 | 9.5 | 10 | 1 | - | Delta | 50 | 1.6 | > 20000^1^ | > 12500 |  |  |  |  |
| 5- Column Comparison (B4) | Batch | B4 | 9.5 | 10 | 1 | - | Delta | 100 | 1.6 | > 20000^1^ | > 12500 |  |  |  |  |
| 5- Column Comparison (B5) | Batch | B5 | 9.5 | 5 | 1 | 1 | Delta | 50 | 1.6 | 7900 | 4938 |  |  |  |  |
| 5- Column Comparison (B5) | Batch | B5 | 9.5 | 5 | 1 | 1 | Delta | 100 | 1.6 | > 20000^1^ | > 12500 |  |  |  |  |
| 5- Column Comparison (A1) | Column | A1 | 9.5 | - | - | - | Delta | 4500 |  |  |  | 5.2E-03 | 4.4E-03 | 84.6% | ND^2^ |
| 5- Column Comparison (A2) | Column | A2 | 9.5 | - | - | 1 | Delta | 4500 |  |  |  | 2.2E-03 | 1.7E-03 | 77.3% | ND^2^ |
| 5- Column Comparison (B1) | Column | B1 | 9.5 | 5 | 0.2 | - | Delta | 4500 |  |  |  | 4.6E-03 | 5.9E-05 | 1.3% | 26.9 |
| 5- Column Comparison (B2i) | Column | B2i | 9.5 | 5 | 1 | - | Delta | 4500 |  |  |  | 8.1E-03 | 1.3E-04 | 1.6% | 33.5 |
| 5- Column Comparison (B2ii) | Column | B2ii | 9.5 | 5 | 1 | - | Delta | 4500 |  |  |  | 3.7E-03 | 1.0E-04 | 2.7% | ND^3^ |
| 5- Column Comparison (B3) | Column | B3 | 9.5 | 5 | 5 | - | Delta | 4500 |  |  |  | 6.8E-03 | 1.0E-04 | 1.5% | 19 |
| 5- Column Comparison (B4) | Column | B4 | 9.5 | 10 | 1 | - | Delta | 4500 |  |  |  | 6.6E-03 | 1.5E-04 | 2.3% | 11.9 |
| 5- Column Comparison (B5) | Column | B5 | 9.5 | 5 | 1 | 1 | Delta | 4500 |  |  |  | 4.3E-03 | 7.3E-05 | 1.7% | ND^3^ |
| *^1^Viscosity values exceeding 20,000 cP could not be measured.* | | | | | | | | | | | | | | | |
| *^2^UCS values were non-detectable (ND) due to a lack of sufficient cohesion.* | | | | | | | | | | | | | | | |
| *^3^UCS values were non-detectable (ND) due to disturbance of specimens from embedded EC sensors.* | | | | | | | | | | | | | | | |
| *^4^All experiments contain 6% by mass colloidal silica.* | | | | | | | | |  |  |  |  |  |  |  |
